# Nutrition training in medical and other health professional schools in West Africa: the need to improve current approaches and enhance training effectiveness

**DOI:** 10.3402/gha.v7.24827

**Published:** 2014-07-30

**Authors:** Roger Sodjinou, William K. Bosu, Nadia Fanou, Lucie Déart, Roland Kupka, Félicité Tchibindat, Shawn Baker

**Affiliations:** 1 UNICEF Regional Office for West and Central Africa, Dakar, Senegal; 2 West Africa Health Organization (WAHO), Bobo-Dioulasso, Burkina Faso; 3 UNICEF Cameroon Country Office, Yaoundé, Cameroon; 4 Bill and Melinda Gates Foundation, Seattle, WA, USA

**Keywords:** nutrition, training, curriculum revision, capacity development, health professional schools, West Africa

## Abstract

**Background:**

Health professionals play a key role in the delivery of nutrition interventions. Improving the quality of nutrition training in health professional schools is vital for building the necessary human resource capacity to implement effective interventions for reducing malnutrition in West Africa. This study was undertaken to assess the current status of nutrition training in medical, nursing and midwifery schools in West Africa.

**Design:**

Data were collected from 127 training programs organized by 52 medical, nursing, and midwifery schools. Using a semi-structured questionnaire, we collected information on the content and distribution of nutrition instruction throughout the curriculum, the number of hours devoted to nutrition, the years of the curriculum in which nutrition was taught, and the prevailing teaching methods. Simple descriptive and bivariate analyses were performed.

**Results:**

Nutrition instruction occurred mostly during the first 2 years for the nursing (84%), midwifery (87%), and nursing assistant (77%) programs and clinical years in medical schools (64%). The total amount of time devoted to nutrition was on average 57, 56, 48, and 28 hours in the medical, nursing, midwifery, and nursing assistant programs, respectively. Nutrition instruction was mostly provided within the framework of a dedicated nutrition course in nursing (78%), midwifery (87%), and nursing assistant programs (100%), whereas it was mainly embedded in other courses in medical schools (46%). Training content was heavily weighted to basic nutrition in the nursing (69%), midwifery (77%), and nursing assistant (100%) programs, while it was oriented toward clinical practice in the medical programs (64%). For all the programs, there was little focus (<6 hours contact time) on public health nutrition. The teaching methods on nutrition training were mostly didactic in all the surveyed schools; however, we found an integrated model in some medical schools (12%). None of the surveyed institutions had a dedicated nutrition faculty. The majority (55%) of the respondents rated nutrition instruction in their institutions as insufficient.

**Conclusions:**

The results of our study reveal important gaps in current approaches to nutrition training in health professional schools in West Africa. Addressing these gaps is critical for the development of a skilled nutrition workforce in the region. Nutrition curricula that provide opportunities to obtain more insights about the basic principles of human nutrition and their application to public health and clinical practice are recommended.

Undernutrition, overweight, and obesity are issues of great concern in developing countries (1). Undernutrition is associated with about 45% of all child deaths globally (2). West Africa is among the regions that are most affected by undernutrition (3). It is estimated that 33% of children under 5 years in West Africa are stunted, 9% wasted, and 20% underweight (4). Four countries in the region (Benin, Liberia, Niger, and Sierra-Leone) are among the countries with the highest stunting prevalence (at least 40%) in the world (4). Further, nearly one million children under five in the West Africa region were admitted in 2013 with severe acute malnutrition (5). Micronutrient deficiencies, mainly vitamin A, iron, zinc, and iodine, are widespread among children and women in the region (1, 6). Of the 20 countries with the highest disease burden attributable to iron, vitamin A, and zinc deficiencies, as measured in disability-adjusted life years (DALYs), nearly half are in West Africa (7). The region is also experiencing a rapid nutrition transition, fueled by rapid urbanization and changes in lifestyle patterns, thus creating a double burden of undernutrition and overnutrition (8, 9). Diet-related chronic diseases are on the rise in West Africa (10).

There is now increasing momentum to improve nutrition in West Africa with countries accelerating their efforts toward reducing child malnutrition. At the time of writing, 15 of the 16 countries in the West Africa region had joined the Scaling Up Nutrition (SUN) movement. The set of interventions that can reduce micronutrient deficiencies and address maternal and child undernutrition are well known (11). However, adequate institutional and human capacity is critical to deliver these interventions (12, 13). A major concern is that the prevailing education system has not kept pace with current and emerging health challenges (14), and so health professionals are not always adequately equipped with the knowledge to tackle nutrition issues. This has led to recent calls for a profound reform in health professionals’ education in developing countries (15). A first step toward this reform is to gain better insights on the current situation of nutrition training in health professional schools.

While the issue of nutrition education in medical and other health schools has been explored quite extensively in developed countries (16–28), it has been scarcely studied in West Africa. We are aware of only one study, conducted in Nigeria, which explored approaches that could be used to introduce nutrition into their medical education curricula (29). The present study was conducted to assess the current status of nutrition training in medical, nursing and midwifery schools in West Africa. The study also aimed to identify any gaps in current approaches to nutrition education in these schools and propose practical solutions to address them.

## Methods

This study is part of a larger assessment of the capacity for action in nutrition in West Africa. The West Africa region is composed of 16 countries, Mauritania and the 15 countries that constitute ECOWAS (The Economic Community of West African States). The study was conducted within the framework of the West Africa Nutrition Capacity Development Initiative (WANCDI), implemented under the auspices of the West Africa Health Organization (WAHO). The details of the sampling procedure and the data collection methods have been described elsewhere (30).

Our study sample included medical, nursing and midwifery schools offering human nutrition courses as part of their training curricula. We used ‘human nutrition’ to refer to all courses or topics that were specifically identified as such in the training curriculum. All participating institutions were fully established and received authorization from their respective governments to run the programs.

A preliminary list of health professional schools offering human nutrition courses as part of their training curricula was compiled based on information provided by in-country key informants and UNICEF country offices. This list was continuously updated until the end of the data collection period.

### Data collection

Using a semi-structured questionnaire (available upon request), administered either to the head of the program or the persons in charge of the nutrition instruction, we collected information about the total number of hours devoted to nutrition across the curriculum, the content and status of nutrition courses, the distribution of nutrition instruction throughout the curriculum, the periods of the curriculum in which nutrition was taught, the prevailing teaching methods, school ownership, institutional affiliation, school location, main funding source, and profile of faculty members involved in nutrition training.

The questionnaire also included a question about the quality of nutrition instruction in the curriculum as perceived by respondents. Data were also collected through literature review or Internet searches.

Three countries (Cape Verde, Guinea-Bissau, and The Gambia) were not included in our sample because our preliminary contacts with in-country key informants revealed that nutrition-training activities were nonexistent or not well developed in tertiary-level institutions or health professional schools. A field visit was conducted to Togo but we did not have the opportunity to discuss with health professional schools in the country

### Data analyses

Data were processed in MS Excel 2010 and later analyzed in SPSS 14.0. Simple descriptive and bivariate analyses were performed. The assessment focused on five major variables: 1) the nutrition content in the training curriculum; 2) the distribution of nutrition instruction throughout the curriculum; 3) the status of nutrition courses; 4) the nutrition instruction time; and 5) the prevailing training delivery methods.

### Nutrition content in the training curriculum

Three main categories served to identify the areas of nutrition emphasized by the various programs: basic nutrition (application of nutrition principles at cellular or organ level), clinical or applied nutrition (application of nutrition principles at individual level), or public health nutrition (application of nutrition principles at population level).

### Distribution of nutrition instruction throughout the curriculum

Three major categories were used to characterize the distribution of nutrition instruction throughout the curriculum: 1) dedicated, stand-alone nutrition course taught in a specific year of the curriculum, 2) integrated nutrition course (nutrition taught as part of other courses), and 3) mixed pattern (dedicated nutrition course and integrated format).

### Status of nutrition courses

Two main categories served to appraise the status of nutrition courses in the curriculum: 1) required nutrition courses and 2) elective nutrition courses.

### Nutrition instruction time

The total nutrition contact time was calculated by adding up the time allocated to dedicated nutrition courses and nutrition instruction taught inside other courses (e.g. pediatrics, cardiology, gynecology, biochemistry, community health, etc.). We were not able to obtain a good estimation of the time allocated for nutrition within other courses for 15 out of the 127 (6%) training programs assessed. For these programs, we divided the time allocated to the entire course by the number of topics covered by the course to get an estimate, thereby assuming that the different topics were of equal duration.

We used the WAHO/ECOWAS recommendations in the harmonized curriculum for nurses and midwives (31) and medical doctors (32) as benchmarks to assess the adequacy of the nutrition instruction time in the different programs. The recommendations were 20 hours of nutrition instruction for nursing and midwifery programs (31) and 120 hours in medical schools (32). There were no specific recommendations for nursing assistant programs.

### Prevailing training delivery methods

The prevailing teaching methods were categorized as didactic method (focus on the acquisition of knowledge), problem-based learning (focus on the acquisition of knowledge, abilities, and skills), and holistic, integrated approach (focus on the acquisition of knowledge, skills, and competencies).

## Ethics and consent

Ethics approval was not needed as the study did not involve data collection on human subjects. All respondents were fully informed about the objectives of the study and gave their full verbal consent before participating.

## Results

### Characteristics of the participating institutions

Data were collected on 127 training programs organized by 52 training institutions, including 11 medical programs, 51 nursing programs, 30 midwifery programs, and 35 nursing assistant programs (Table 1).

**Table 1:** Number of participating institutions and training programs assessed^a^

		Number of training programs assessed			
		
Country	Number of participating institutions	Medicine	Nursing	Midwifery	Nursing assistant
Benin	2	2	0	0	0
Burkina-Faso	6	2	6	4	10
Cote-d'Ivoire	1	0	1	1	0
Ghana	1	1	0	0	0
Guinea	6	0	4	5	0
Liberia	1	0	1	0	0
Mali	7	1	1	2	8
Mauritania	4	1	8	4	0
Niger	8	1	9	7	10
Nigeria	3	1	2	0	0
Senegal	9	2	8	7	7
Sierra-Leone	4	0	11	0	0
Total	52	11	51	30	35

a Four countries in West Africa (Cape Verde, Guinea-Bissau, and The Gambia) were not included in our study.

**Fig. 1:**
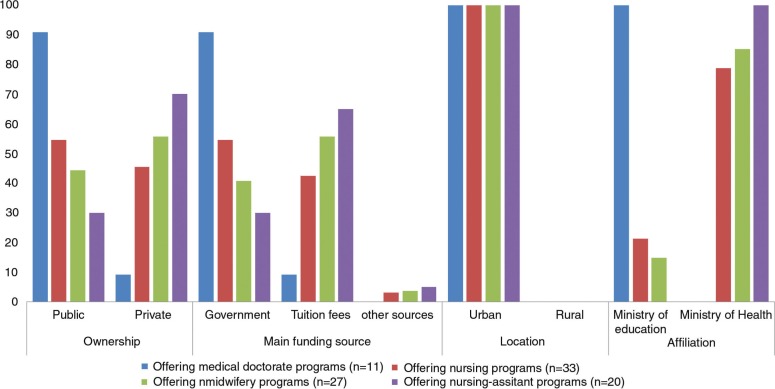
Characteristics of participating institutions^a^ (*n*=52). ^a^Note that there were institutions offering more than one training program.

The majority of the medical (91%) and nursing (55%) schools were public, government-owned institutions (Fig. 1) while the majority of midwifery (56%) and nursing assistant (70%) schools were privately owned. Correspondingly, the majority of medical (91%) and nursing (55%) schools relied mostly on government subsidies as their main source of funding, whereas midwifery (57%) and nursing assistant (65%) schools relied mostly on tuition fees (Fig. 1).

All the medical schools were supervised by the Ministry of Education, whereas majority of the nursing (79%), midwifery (85%), and nursing assistant (100%) schools were supervised by the Ministry of Health (Fig. 1). All the participating institutions were located in urban areas (Fig. 1).

### Nutrition contact time

Nutrition instruction occurred mostly during the first cycle (first 2 years) for the nursing (84% of the programs), midwifery (87%), and nursing assistant (77%) programs (Table 2). Conversely, nutrition was mostly (64%) taught during clinical years (second cycle) in medical schools.

**Table 2:** Nutrition instruction year and contact time

			Medical programs (*n*=11)	Nursing programs (*n*=51)	Midwifery programs (*n*=30)	Nursing assistant programs (*n*=35)
Curriculum year^a^ (%)	First cycle only		9.1	84.3	86.6	77.1
	Second cycle only		63.6	2	6.3	22.9
	Throughout the curriculum		27.3	7.8	0	0
	Other patterns^b^		0	5.9	7.1	0
Areas emphasized	Basic nutrition	Number of programs (%)	4 (36.4)	35 (68.6)	23 (76.7)	35 (100)
in the curriculum		Hours of contact^c^	15.8±6.8	41.9±3.1	40.4±3.6	28.0±2.1
	Applied nutrition	Number of programs (%)	7 (63.8)	10 (19.6)	5 (16.7)	0
		Hours of contact^c^	37.5±10.9	9.6±2.6	6.2±2.3	0
	Public health	Number of programs (%)	0	6 (11.8)	2 (6.6)	0
	nutrition	Hours of contact^c^	3.6±2.4	5.0±1.7	2.9±1.3	0
Average hours of contact^c^			56.9±5.7	56.4±4.0	48.3±3.3	28.0±2.1
Range (hours)			28–92	20–120	20–110	08–70

a Studies for the doctoral programs consist generally of a first cycle (first 2 years or pre-clinical years), a second cycle (clinical years) followed by a third cycle (covering four semesters in the Francophone countries and the period of housemanship in Anglophone countries). Nursing and midwifery schools have two cycles (pre-clinical and clinical years).

b This includes courses taught during years 1 and 3 or years 2 and 3 for the nursing and midwifery programs or during year 3–7 for the medical programs.

c Values are expressed as mean ± standard error.

Based on available data, we estimated that the total number of hours devoted to nutrition in medical, nursing, midwifery, and nursing assistant schools was on average 57.0 hours (range: 28–92), 56.4 hours (range: 20–120), 48.3 hours (range: 20–110), and 28.0 hours (range: 8–70), respectively (Table 2).

All of the nursing and midwifery programs met the WAHO/ECOWAS recommendation of 20 hours of nutrition instruction. In contrast, none of the medical schools met the WAHO/ECOWAS recommendations of 120 hours of nutrition contact time.

### Nutrition areas emphasized and topics covered

Nutrition instruction was heavily weighted to basic nutrition in the nursing (69%), midwifery (77%), and nursing assistant (100%) programs (Table 2). The amount of time devoted to basic nutrition was on average 42 hours (74% of the total nutrition contact time), 40 hours (84%), and 28 hours (100%), respectively. In contrast, nutrition instruction was mostly (64%) oriented toward clinical practice in the medical curricula, with an average of 38 hours (67%) for clinical nutrition. For all the programs, there was less than 6 hours of contact time used on public health nutrition.

In general, topics covered in basic nutrition courses included physiological aspects of nutrition, nutritional biochemistry, body composition, and nutrition assessment. In medical schools, nutrition was generally taught mostly within several courses such as pediatrics and biochemistry.

### Structure and distribution of nutrition instruction

Nutrition instruction was either provided within the framework of a required, dedicated nutrition course or integrated with other courses in the curriculum (Table 3). Some schools used both approaches. None of the schools offered elective nutrition courses.

**Table 3:** Characteristics of the nutrition training curricula of the health programs

Characteristic		Medical (*n*=11)	Nursing (*n*=51)	Midwifery (*n*=30)	Nursing assistant (*n*=35)
Distribution of nutrition instruction throughout the curriculum (%)	Dedicated nutrition courses	27	78	87	100
	Integrated with other courses	46	4	0	0
	Both patterns	27	18	13	0
Teaching format (%)	Didactic	88	100	100	100
	Problem-based learning	0	0	0	0
	Integrated system-based	12	0	0	0
Status of nutrition courses (%)	Required	100	100	100	100
	Elective	0	0	0	0

We found that nutrition was mostly taught as a dedicated course for the nursing (78%), midwifery (87%) and nursing assistant (100%) programs (Table 3). In contrast, nutrition instruction was mainly (46%) embedded in other clinical courses in the medical programs.

### Teaching format

The teaching methods in nutrition education used for all the programs were mostly very didactic, with little emphasis on the practical applications of nutrition principles (Table 3). However, we found integrated, system-based nutrition curriculum in 2 out of the 11 (12%) medical schools.

### Faculty members

None of the surveyed institutions had a full-time, dedicated faculty member with expertise in nutrition. The faculty members who taught nutrition were either external, part-time nutrition faculty contracted by the schools or internal faculty from other disciplines.

### Quality of nutrition instruction as perceived by respondents

Of 31 respondents, 55% rated the quality of nutrition instruction in their institutions as inadequate or insufficient while 35% rated it as adequate. The remaining 10% offered no comments. All the respondents expressed the need to enhance current approaches to nutrition education to better prepare graduates to perform nutrition-care tasks.

## Discussion

In this study, we assessed the current status of nutrition training in medical, nursing, and midwifery schools in West Africa. Our results showed that all the surveyed institutions devoted at least 20 hours to nutrition instruction, which attests to the awareness on the importance of nutrition as a key component of health professionals’ education. We found that students in medical and nursing schools received more nutrition training than midwifery and nursing assistant students. This may be due in part to the fact that medical doctors and registered nurses provide the bulk of nutrition interventions in West Africa.

We also found that medical programs are teaching less nutrition than what is recommended by WAHO/ECOWAS. This situation may be partly explained by the fact that the WAHO/ECOWAS harmonized curriculum guideline was released only recently in March 2013 and so the surveyed countries had not had sufficient time to implement it. In 2012, Nigeria also adopted a national template for the Bachelor of Medicine, Bachelor of Surgery (MB BS) curriculum, with an emphasis on human nutrition (33). Other countries such as Ghana and Mauritania are also in the process of reviewing their training curricula. With all these forthcoming changes in the region, it is expected that nutrition instruction in medical schools will be gradually enhanced.

Conversely, we found that all of the nursing and midwifery schools met the recommendation of 20 hours of nutrition instruction. This is probably due to the low threshold level proposed by WAHO/ECOWAS. We believe that 20 hours of nutrition instruction may not be enough to cover all domains of human nutrition. Nutrition is a vast discipline covering a wide range of topics and issues from the molecular to population levels. It would be important to revise the WAHO/ECOWAS recommendations on nutrition instruction in nursing and midwifery schools. However, it is not our intention in this paper to make specific recommendations on the minimum number of hours of nutrition that should be taught in these schools. WAHO has several platforms with national and regional bodies with whom this issue and the larger issue of standards could be discussed.

Nutrition instruction occurred mostly within the framework of a dedicated nutrition course in nursing, midwifery and nursing assistant programs. The topics covered were mainly on basic nutrition and training methods were very didactic. There are several reasons that may explain why current nutrition curricula are heavily weighted to basic science. First, nutrition instruction occurs mostly during the first 2 years of the curriculum, where the focus is on didactic teaching. Similar observations have been made in previous studies (16, 17). Second, all the surveyed schools are located in urban areas. This likely limits the opportunities for students to practice in rural communities, where the vast majority of nutrition interventions are implemented. Third, the lack of dedicated teaching staff with expertise in nutrition could affect the capacity of institutions to link basic nutrition to clinical practice. Several studies recommend training more teaching staff in nutrition in medical schools (16, 19, 21, 28). Although it is important for students to have a good understanding on the basic principles of nutrition, it is even more critical for them to gain better insights on their application to clinical practice (16–19, 21, 23–27).

Unlike the situation in the nursing and midwifery schools, nutrition education in medical schools was oriented toward clinical practice with students receiving little training in basic nutrition. This means that they may not have the needed background knowledge to understand the application of nutrition to public health or clinical practice. Until recently, nutrition training at undergraduate level was not part of the academic system in the francophone countries of West Africa (30). With only a few registered dieticians available in these countries, health professionals have been the main interface for counseling patients on dietary practices. They therefore need sound knowledge on basic nutrition to be able to provide dietary recommendations that would help prevent nutrition-related chronic diseases or lifestyle modification-related counseling to patients with diabetes, hypertension or cardiovascular diseases. Similarly, medical doctors and nurses need to have a good understanding of the scientific basis of the protocol for integrated management of acute malnutrition and its application in clinical settings. Problem-based learning, an approach that builds on synergies between basic science and clinical practice, can be a good way to link basic nutrition to clinical practice (18).

Overall, there is a need to reshape nutrition training in health professional schools in West Africa. A preliminary step in moving toward this direction would be to focus on the distribution of nutrition courses throughout existing curricula rather than to increase the nutrition instruction time (16, 19). Given the fact that current curricula are already overloaded, recommending an increase in nutrition instruction time is not likely to be accepted by health professional schools in West Africa. Many authors recommend a multifaceted nutrition-training curriculum that provides better insights on the basic principles of nutrition and their application to clinical practice (16, 18, 19, 23). There is also a need for an initial dedicated nutrition course supplemented by a comprehensive integration of nutrition throughout the curriculum (16). All these recommendations could be adapted to the West African context. In Table 4, we have identified a minimum set of competencies and topics that could be used as a basis to enhance nutrition training in health professional schools in West Africa and prepare students to tackle real-life nutrition challenges. Most of these topics are part of the critical nutrition interventions highlighted in the recent Lancet series on maternal and child malnutrition (1).

**Table 4:** Core competencies and possible topics to be included in the curriculum of students in health professional schools

Status of nutrition course^a^	Year in the curriculum^a^	Focus area	Teaching method	Competencies and topics	Medical programs^b^	Nursing and midwifery programs^b^
Integration of didactic, public health-oriented and applied nutrition courses throughout the curriculum	Throughout the curriculum, from pre-clinical to clinical years	Basic nutrition	Didactic	Core competencies	Be able to understand the basic principles of human nutrition, assess the nutritional status of individuals and groups, and conduct dietary assessment	
				Possible topics	Nutrition biochemistry Nutritional requirements Nutritional physiology Dietary and nutritional assessment Food composition Micronutrient deficiencies Infant and young child feeding	
		Clinical nutrition	Integrated, system-based format	Core competencies	Be able to manage acute malnutrition using the national protocol, manage micronutrient deficiencies, and provide dietary and lifestyle advices for the management of obesity and diet-related chronic diseases	Be able to manage acute malnutrition using the national protocol, provide nursing care for the management of micronutrient deficiencies and diet-related chronic diseases
				Courses in which nutrition topics can be integrated	Pediatrics Endocrinology Clinical medicine	Pediatrics Clinical practices focused on acute disease management Clinical practices focused on chronic disease management Nursing care Dietetics Obstetrics and gynecology
					Multidisciplinary health care delivery Family medicine Preventive medicine Digestive system Cardiovascular disease	
		Public health nutrition	Problem-based learning and/or Integrated, system-based format	Core competencies	Be able to promote appropriate IYCF, prevent micronutrient deficiencies and diet-related chronic diseases through the application of public health principles; Be able to manage nutrition in emergencies	
				Possible topics	Nutrition and health promotion Nutrition in emergencies Food and nutrition policies Community nutrition Food systems, nutrition and health	

a Adapted from Refs. 20, 22, and 25.

b Some of the topics were derived from Ref. 1.

There are several noteworthy limitations to this study. First, we did not receive answers to all questions of the survey questionnaire. Second, the proxy method used, in some cases, to estimate the instruction time for nutrition taught within other courses might underestimate or overestimate the actual nutrition instruction time. However, we believe that its impact on our findings is little as this estimation was done for only 6% of the training programs assessed. Moreover, the bulk of nutrition instruction is done within dedicated nutrition courses. Finally, we were not able to cover all of the medical and nursing schools in West Africa. However, we believe the conclusions reached in this study remain relevant owing to the common challenges of nutrition training in West Africa.

Our study has several strengths. This is the first-ever region-wide assessment of the status of nutrition training in health professional school in West Africa. Our findings offer the opportunity to revisit the current recommendations for nutrition instruction in health professional schools in the region. They serve as a guide in developing a regional strategy for enhancing nutrition training in health professional schools in West Africa.

## Conclusions

Our findings reveal important gaps in the amount of instruction time, distribution of the nutrition instruction in the health curricula, teaching methods and the link between basic nutrition and clinical and public health nutrition. A comprehensive coverage of all essential aspects of human nutrition in health professional schools in West Africa is needed. In addition, nutrition instruction needs to occur throughout the training curriculum. Teaching approaches should enable students to apply the basic principles of human nutrition to public health and clinical practice.
